# Enhancing Performance of Magnetic Field Based Indoor Localization Using Magnetic Patterns from Multiple Smartphones

**DOI:** 10.3390/s20092704

**Published:** 2020-05-09

**Authors:** Imran Ashraf, Soojung Hur, Yongwan Park

**Affiliations:** Department of Information and Communication Engineering, Yeungnam University, Gyeongbuk, Gyeongsan-si 38541, Korea; ashrafimran@live.com (I.A.); sjheo@ynu.ac.kr (S.H.)

**Keywords:** Indoor localization, magnetic field-based localization, smartphone sensors, pattern matching, fingerprinting, pedestrian dead reckoning

## Abstract

Wide expansion of smartphones triggered a rapid demand for precise localization that can meet the requirements of location-based services. Although the global positioning system is widely used for outdoor positioning, it cannot provide the same accuracy for the indoor. As a result, many alternative indoor positioning technologies like Wi-Fi, Bluetooth Low Energy (BLE), and geomagnetic field localization have been investigated during the last few years. Today smartphones possess a rich variety of embedded sensors like accelerometer, gyroscope, and magnetometer that can facilitate estimating the current location of the user. Traditional geomagnetic field-based fingerprint localization, although it shows promising results, it is limited by the fact that various smartphones have embedded magnetic sensors from different manufacturers and the magnetic field strength that is measured from these smartphones vary significantly. Consequently, the localization performance from various smartphones is different even when the same localization approach is used. So devising an approach that can provide similar performance with various smartphones is a big challenge. Contrary to previous works that build the fingerprint database from the geomagnetic field data of a single smartphone, this study proposes using the geomagnetic field data collected from multiple smartphones to make the geomagnetic field pattern (MP) database. Many experiments are carried out to analyze the performance of the proposed approach with various smartphones. Additionally, a lightweight threshold technique is proposed that can detect user motion using the acceleration data. Results demonstrate that the localization performance for four different smartphones is almost identical when tested with the database made using the magnetic field data from multiple smartphones than that of which considers the magnetic field data from only one smartphone. Moreover, the performance comparison with previous research indicates that the overall performance of smartphones is improved.

## 1. Introduction

The last decade observed a wide expansion of smart devices, a major part of which comprises smartphones. As a consequence, many smartphone centered services have emerged, e.g., Location Based Services (LBS). LBS has a substantial share of the consumer market and would continue to grow in the coming years. LBS services are offered for both indoor and outdoor environments and precise location information serves as the backbone of such services. Global Positioning System (GPS) is one of the most widely used outdoor localization techniques and provides a meter level accuracy [1]. For the indoor environment, GPS can provide the user’s location especially if he is near large glass windows, the provided accuracy may not be enough to serve the indoor LBS. GPS’s indoor localization error maybe even larger than the indoor space itself in many cases. For this reason, alternative indoor localization technologies have been investigated and proposed like Radio Frequency Identification (RFID) [2,3], Ultra Wide Band (UWB), Wi-Fi [4] and Bluetooth Low Energy (BLE) [5]. These techniques, however, require the installation of additional infrastructure in the form of sensors. Besides, there are other limitations associated with each of these technologies. For example, RFID needs active or passive tags with the localization environment or the user and a scanner [6,7] to perform the localization while UWB need tags/anchors to locate the user. Ubisense is a well-known positioning system that uses UWB. The user carried tags transmit signals while the fixed sensors can locate the user using the time of arrival technique [8]. Similarly, BLE localization involves placing Beacons at various positions/landmarks and works both with received signal strength and channel state information [9,10]. Although Wi-Fi based localization does not require additional hardware and utilizes vastly deployed Wireless Access Points (WAP), yet often show poor performance under dynamic conditions where the radio signals suffer from shadowing and multi-path in crowded areas like shopping malls, train stations, and airports, etc.

Recently the availability of a large range of micro-electromechanical system (MEMS) sensors embedded in smartphones provided new opportunities to perform the localization without the installation of additional infrastructure. These sensors though small in size offer sufficient precision to perform indoor localization. So, many approaches utilizing the data from smartphone sensors including accelerometer, gyroscope, and magnetometer have been proposed during the last few years [11,12,13]. Pedestrian Dean Reckoning (PDR) based localization provides a relative location and requires a starting or recent position of the user. Magnetometer, on the other hand, measures the earth’s natural magnetic field (referred to as ‘magnetic field’ in the rest of the paper) and works similar to Received Signal Strength (RSS) based Wi-Fi systems. It neither requires any additional infrastructure nor needs the starting position of the user. Although magnetic field-based localization systems offer a good level of accuracy, their performance is often degraded by the use of a large variety of smartphones that are available today. Smartphones’ companies install the magnetometers from various vendors, each with a different level of noise tolerance and sensitivity, which severely limits the full potential of such systems. Various improved techniques have been presented where the use of patterns formed by the magnetic field data (called Magnetic Pattern (MP) for simplicity) is preferred over the magnetic intensity to overcome the limitation of device dependency, yet, they are still limited by the procedure adopted to make the database [14,15]. It is argued that the MP from various smartphones is similar, however, this is not very well studied.

We aim to investigate the above-mentioned problem in detail and provide an in-depth analysis of MP from various smartphones. After a comprehensive analysis, it is found that the MP from various smartphones are not the same which leads to different localization performance of smartphones even when the very same localization approach is used. We, therefore, propose the use of the data from multiple smartphones to make the MP database to mitigate the impact of device dependency. Specifically, this paper makes the following contributions:

- A detailed analysis of magnetic field data from a variety of smartphones is made to study the differences in magnetic field patterns.
- A comprehensive investigation is done to explore the feasibility of using the magnetic field patterns from multiple smartphones to make the database for indoor localization.
- The performance of an Artificial Neural Network (ANN) is compared against a threshold-based motion detection module as well as Decision Trees (DT), Classification And Regression Trees (CART), Naive Bayes (NB) and K Nearest Neighbor (KNN). The motion detection module serves as an important element of indoor localization systems.
- An indoor localization approach is proposed which utilizes the MP of the magnetic field data to reduce the impact of device dependency on the magnetic field-based indoor localization.
- A detailed analysis is done on the individual use of various smartphones for making the database and its impact on localization performance.

The rest of the paper is organized in the following manner. Section 2 discusses research works that are closely related to the current study. Section 3 describes the proposed approach for localization, as well as, the evaluation of various motion detection techniques like threshold-based, ANN, and DT, etc. Results are presented in Section 4 while the conclusion and limitations are given in Section 5.

## 2. Overview of Magnetic Field and Magnetic Positioning Approaches

The magnetic field is the earth’s natural phenomenon generated by the flow of convection current in the earth’s outer layer. Being a vector (it has direction and magnitude), the magnetic field needs three parameters for its representation. A common approach is to use *x*, *y*, and *z* which indicate the north, east, and downward components of the magnetic field, respectively. An alternative approach is to show the magnetic field through the total intensity *F*, the inclination angle *I*, and the declination angle *D* [16]. From a magnetic positioning perspective, the magnetic field is often represented with *x*, *y*, *z*, and *F*. The magnetic intensity of the magnetic field differs from 25 micro Tesla to 65 micro Tesla [17] and its magnitude and direction remain approximately the same over a small restricted area. Despite that, man-made buildings interfere with the natural magnetic field and cause disturbances called ‘anomalies’, which have been utilized as fingerprints in many approaches [18,19].

Predominantly, most approaches are based on the fingerprinting database where the magnetic *x*, *y*, and *z* are used to populate the database, as using one magnetic element has limited accuracy. Authors in [20] investigated the use of single vs multiple magnetic elements from a smartphone magnetometer to perform indoor localization. They conclude that the localization accuracy can be improved if more elements of the magnetic field are used. An additional finding is the impact of the area on localization accuracy, whereby the error can go up to 20 m within a larger area. Similarly, an indoor localization approach is introduced in [21] which builds the magnetic map using Local Weight Regression (LWR) presented by Cleveland [22]. The LWR leverages local data to fit points using polynomial weighted fitting where the polynomial coefficient is calculated through the least square method. The localization accuracy is under 1 m within a small building of 27 × 7 m${}^{2}$ dimensions.

Fingerprinting involves an offline phase of data collection which consumes a large amount of time, so various substitutes are offered like crowd-sourcing. For example, authors in [23] proposed fingerprinting based navigation which functions on the crowdsourced built magnetic map. A revised Monte Carlo localization approach is adopted to locate the user. Initially, approximately 30 s data is used to estimate the starting position of the user. Although the proposed algorithm can converge 90% of the time to within 5 m, the amount of data used for location estimation is large.

Accuracy for magnetic fingerprint-based positioning can be enhanced with several alternate strategies. For example authors in [24] propose the use of magnetic landmarks to improve the localization accuracy. The database is made using magnetic *x*, *y*, and *z* components of the magnetic field which is then clustered using the expectation-maximization algorithm. Each cluster serves as a unique landmark that is used to find the location of the user. Another possibility is to make use of data from multifarious sensors like W-Fi, accelerometer, BLE, etc., and develop a hybrid system. For example, an approach based on sensor fusion is presented in [25] that exploits the data from WiFi, smartphone camera, magnetometer, Bluetooth, and people co-occurrence. Camera image helps to narrow down the search space and Wi-Fi can function periodically to correct location estimation. Experimental results indicate that an 83.7% accuracy can be achieved to locate the user with the proposed approach. Additionally, user time-specific activities are also very helpful to determine his location.

The aforementioned works are limited by one or more of the following factors. The use of Wi-Fi and BLE is not appropriate regarding their dependence on the deployed APs and Beacons, as well as, the process of AP and node scanning which drains the smartphone battery. Moreover, Android 9 (Pie) introduced a Wi-Fi scan throttling that restricts the Wi-Fi scanning frequency of the smartphones. It allows four scans in a 2 min period for foreground apps and one scan in 30 min for background apps [26]. The use of camera images involves machine and deep learning approaches that cannot be deployed on the smartphone as a server is needed with a communication link to the smartphone. In addition to that, the majority of the above-cited works are tested with a single smartphone, and the device dependence is not very well investigated. There are only a few research works, where the MP is used to reduce the influence of various smartphones on localization accuracy. For example authors in [27] prefer the use of MP over the magnetic intensity to reduce device dependency. However, in the proposed approach they fuse the Wi-Fi data with the magnetic field data using an augmented particle filter to increase accuracy. Further, only one smartphone is tested with the proposed approach. Similarly, other research works [14,28] selected the use of MP over the intensity data to evaluate the performance of two different smartphones. Although the impact of various smartphones is minimized, the localization performance from various smartphones is largely different. Hence, this research proposes the use of MP built from the magnetic data of multiple smartphones, contrary to previous approaches, who utilized the data from a single smartphone.

## 3. Materials and Methods

The method proposed in this study comprised of different modules that are discussed separately in the following sections.

### 3.1. Motion Detection using Accelerometer

The first and foremost part of the proposed method is to determine the user’s walking and stationary states. It is very important to predict an accurate state as it not only improves the localization accuracy but can save smartphone battery as well. Various techniques have been utilized for the said task including machine learning classifiers like NB, Random Forest (RF), extra tree classifier and ANN, etc. [29]. The use of ANN has been reported to produce more accurate results than that of traditional machine learning methods like NB, and RF, etc., in many research works [14,30,31,32]. However many factors make the use of ANN inappropriate for smartphone-based indoor localization. First of all, it requires a large amount of data for training and validation and smaller datasets can decrease its performance [33,34]. Secondly, resources required for ANN training are yet not supported by the smartphone, so, the training is carried out on a computer. Thirdly, even when trained on a computer it is not possible to deploy it on a smartphone, not for at least now. So, it requires two additional units for real-time localization; a server where the trained ANN-model is available and a channel for the communication between the user smartphone and the server. It also introduces the latency depending upon the type of channel used for communication. Similarly, other machine learning methods, although, not highly computing resources hungry, are limited by similar constraints. For this purpose, this study investigates the use of a threshold method where the accelerometer data from a smartphone is utilized for user motion detection. It is no secret that ANN and other machine learning techniques show superior performance in motion detection tasks, yet, the objective here is to evaluate, how closer a threshold method can be to the accuracy offered by machine and ANN methods.

**Figure 1:**
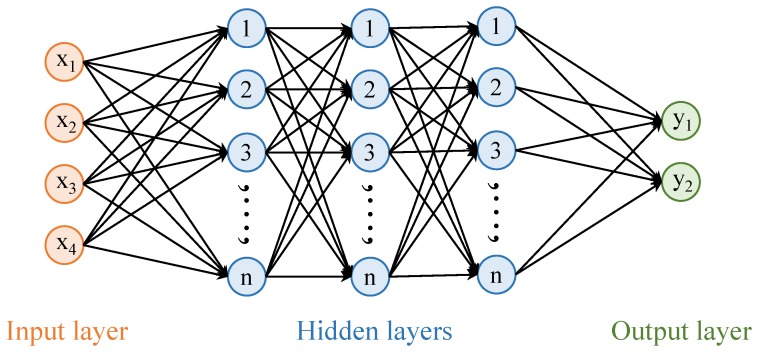
Architecture of artificial neural network used for motion detection.

Towards this end, four of the most widely used machine learning classifiers have been investigated like DT, CART, NB, and KNN. DT is a simple, yet powerful tool to infer decisions from a set of features. DT is comprised of the root nodes, the internal nodes, and the terminals, where nodes and edges are the representatives of features and decision, respectively [35]. DT is favorable because it is non-parametric and computationally inexpensive. Results from DT are easy to interpret and it can tolerate the redundant attributes in the data. CART is intuitive to easily visualize the predictors and can work with numeric, binary, and categorical data. It is noise-tolerant and insensitive to missing values as it can accommodate the missing data with surrogates [36,37]. It recursively splits the data into groups and grows the decision tree until a user-defined threshold is satisfied. The overfitting can be avoided by making a trade-off between the number of terminal nodes and deviance. Based on the Bayesian theorem, NB can predict the probability of a particular sample to a specific class. NB is simple, yet often more effective than other sophisticated classifiers [38,39]. Assuming that the values of attributes are conditionally independent, it can assign the sample to a class that achieves the highest posterior probability. KNN is one of the most widely used classifiers which is simple yet efficient by its structure. Often called ‘lazy learner’, it does not make any assumptions about the data distribution. Given *k* neighbors, it divides the samples into different classes by deriving boundaries between the classes. Various choices for distance estimation between data points are considered, and Euclidean Distance (ED) has been regarded as a good choice for numerical data points. A new sample is attributed to a particular class based on the voting of its neighbors [40]. The ANN with the structure shown in Figure 1 is used for motion detection.

ANN used in this study has three hidden layers with ten neurons each. Hidden layers are fully connected and the stochastic gradient descent method is used for optimization. A total of one hundred epochs are used for training whereas the train test split is 80–20 and the learning rate is set to 0.01. The task of ANN is to predict the samples into motion and stationary classes and feature vector is comprised of four features as shown in Table 1.

Before calculating the features from the accelerometer data, two important processes are carried out: bias correction and noise removal. Bias is the error in the acceleration data even after the accelerometer is calibrated. It needs to be estimated and removed. For this purpose, the smartphone is put motionless on a plain surface and the acceleration in *x*, *y*, and *z* is noted. Any difference in the acceleration from 0, 0 and 1 g (9.8 m/s${}^{2}$) for *x*, *y*, and *z* acceleration needs to be adjusted. So, the bias-free acceleration can be estimated as

(1) $$a_{x}^{c}=a_{x}^{m}-S\times a_{x}^{a}$$

where $a_{x}^{c}$, $a_{x}^{m}$ and $a_{x}^{a}$ represent the corrected, measured and actual acceleration for *x* axis.

Using the corrected acceleration for *x*, *y*, and *z*, the total corrected acceleration can be calculated as

(2) $$A^{c}=\sqrt{{\left(a_{x}^{c}\right)}^{2}+{\left(a_{y}^{c}\right)}^{2}+{\left(a_{z}^{c}\right)}^{2}}$$

**Figure 2:**
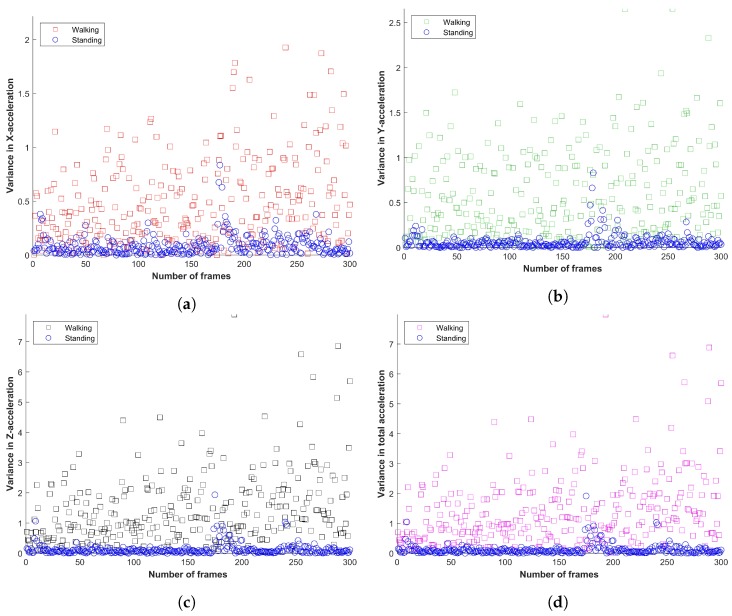
Variance in smartphone accelerometer data; (**a**) *x*-axis acceleration, (**b**) *y*-axis acceleration, (**c**) *z*-axis acceleration and (**d**) total acceleration.

Features selected for user’s state detection are selected due to their variability when the user is either walking or standing still. Of course, it is possible to fetch derived features from accelerometer data like mean, median, and inter-quartiles, etc. however it increases the feature vector and requires increased training time and resources. Instead, this study considers only the acceleration in *x*, *y*, *z*, and total acceleration for user motion detection. The attitude of the selected features for walking and standing motionless is shown in Figure 2.

The same features are used for threshold-based motion detection. Two threshold scenarios are investigated and called $T_{1}$ and $T_{1}$. The goal is to refine the threshold values to detect users’ states of motion and stationary. A two-step procedure is adopted for this purpose;

1. Find a threshold α for each feature that can individually detect user motion.
2. Find a combination of such αs for scenarios $T_{1}$ and $T_{2}$ to refine the motion detection accuracy.

In $T_{1}$ the threshold of variances is joined through ‘AND’ while for $T_{2}$ the individual variances are joined using ‘OR’. The latter case is simple where the initially estimated individual variances are joined, while the former involves the adjustment which is done by varying the individual variances with a δ value. The value for δ is 0.01 and it is both added, as well as, subtracted from individual variances to find an optimal α for *x*, *y*, *z*, and *a* variance for motion detection.

### 3.2. Step Detection and Heading Estimation

**Figure 3:**
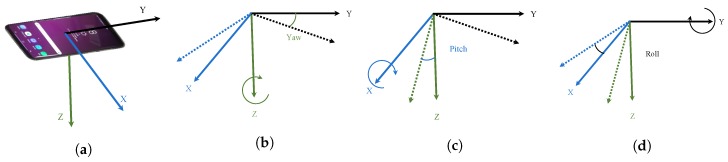
Rotation of smartphone in 3D orientation, (**a**) Smartphone coordinate system, (**b**) rotation around *z*-axis (yaw), (**c**) rotation around *y*-axis (pitch), and (**d**) rotation around *x*-axis (roll).

Step detection and heading estimation are performed using the accelerometer and gyroscope data from smartphone sensors. The bias correction for accelerometer and gyroscope is carried using the procedure given in Equation (1). Later, a low pass filter is used to remove noise in the data before further processing.Euler angles are used to transform smartphone motion to the inertial frame. There are three kinds of rotation for a smartphone as shown in Figure 3.

For reproducibility, this section discusses the coordinate transformation and yaw calculation as they are implemented in Android Studio 3.5. Coordinate transformation and yaw calculation require the data from three sensors: the magnetometer, accelerometer, and gyroscope (represented as M, A, and G, respectively). The sensor manager used in Android is represented as SM. First, a rotation matrix *R* is obtained using M and G. *R* corresponds to a 3 × 3 matrix as follows:

(3) $$R=\left[\begin{array}{ccc} r_{0} & r_{1} & r_{2}\\ r_{3} & r_{4} & r_{5}\\ r_{6} & r_{7} & r_{8} \end{array}\right]$$

In Android, it is obtained using acceleration and magnetometer data as follows:

(4) SM.getRotationMatrix(R,null,A,M)

*R* is used to get the orientation angles, which corresponds to a 3 × 1 matrix as follows:

(5) $$O=\left[\begin{array}{c} o_{0}\\ o_{1}\\ o_{2} \end{array}\right]$$

In Android, *O* is obtained using *R* as follows:

(6) SM.getOrientation(R,O)

The elements of *O* are ϕ, θ, and ψ at 2, 1, and 0 indices, respectively. However, the orientation angles and gyroscope data need to be integrated over the change in time, represented here as dT. This is done in Android as follows:

(7) $$\left[\begin{array}{c} \phi \\ \theta \\ \psi \end{array}\right]=\left[\begin{array}{c} o_{0}\times dT\\ o_{1}\times dT\\ o_{2}\times dT \end{array}\right]$$

(8) $$\left[\begin{array}{c} gyr_{0}\\ gyr_{1}\\ gyr_{2} \end{array}\right]=\left[\begin{array}{c} G_{0}\times dT\\ G_{1}\times dT\\ G_{2}\times dT \end{array}\right]$$

Later, ϕ, θ, and ψ are used to calculate the Euler angles *E*. Euler angles correspond to a 3 × 3 matrix and are calculated in Android as follows:

(9) $$E=\left[\begin{array}{ccc} 1 & sin(\phi )\times tan(\theta ) & cos(\phi )\times tan(\theta )\\ 0 & cos(\phi ) & -sin(\phi )\\ 0 & sin(\phi )/cos(\theta ) & cos(\phi )/cos(\theta ) \end{array}\right]$$

The user walking angle (Ang) is obtained using the Euler angles and integrated gyroscope data gyr calculated in Equation (8). It is calculated using

(10) $$Ang=E\left[2\right]\left[0\right]\times gyro_{0}+E\left[2\right]\left[1\right]\times gyro_{1}+E\left[2\right]\left[2\right]\times gyro_{2}$$

The $\ddot{\psi }$ represents the change in user, direction and can be obtained by subtracting the previous angle (called the baseAngle) from Ang. The baseAngle is replaced with Ang every time a new calculation is made. Then, $\ddot{\psi }$ can be used with the user’s step and step length estimation to estimate their current relative position.

Step detection is carried out with the algorithm proposed in [14], and step length estimation is done using the Weinberg model [41]:

(11) $$S_{l}=k\sqrt[{4}]{a_{max}-a_{min}}$$

where $a_{max}$ and $a_{min}$ are the maximum and minimum acceleration in the given acceleration and *k* is a threshold calculated during the calibration phase. The value of *k* used in this study is 0.435. Once $S_{l}$ and the number of steps $S_{n}$ found in a given time *t* (2 s) are calculated, user position can be estimated as:

(12) $$\begin{array}{c} x_{i}=x_{i-1}+S_{n}\times S_{l}\times \cos\left({\ddot{\psi }}_{i-1}\right) \end{array}$$

(13) $$\begin{array}{c} y_{i}=y_{i-1}+S_{n}\times S_{l}\times \sin\left({\ddot{\psi }}_{i-1}\right) \end{array}$$

**Figure 4:**
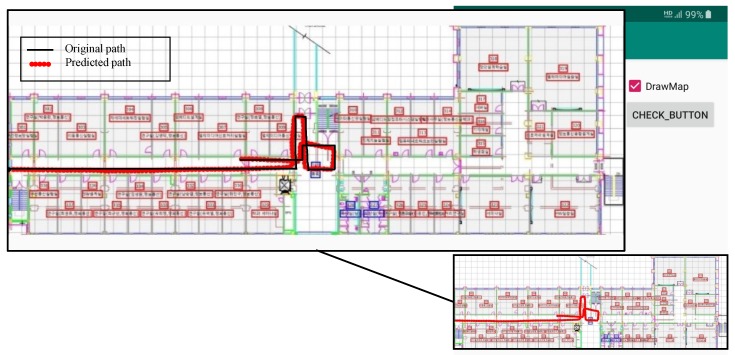
Direction estimation and distance estimation using the developed application for path geometry 1. The enlarged part of the screenshot shows the original and predicted paths.

**Figure 5:**
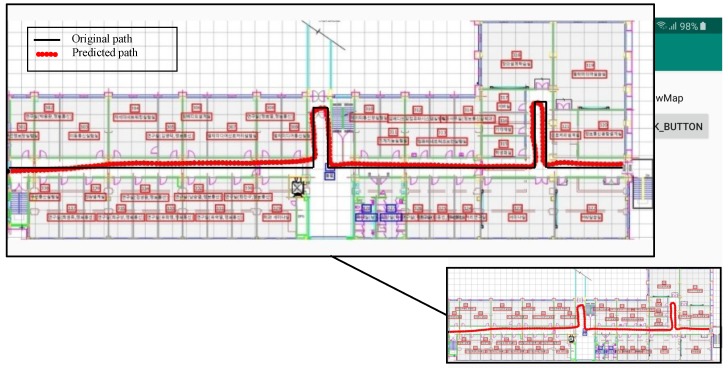
Direction estimation and distance estimation using the developed Android application for path geometry 2. The predicted path shows continuous distance and heading estimation using only Pedestrian Dean Reckoning (PDR), and hence deviations can be found. However, such deviations can be corrected once merged with the magnetic data.

Figure 4 and Figure 5 show the screenshots from the Android application for the predicted path for two different geometries.

Results shown in Figure 4 and Figure 5 indicate only the output of the PDR module and do not portray the localization results. It is obvious from the figures that the gyroscope error is accumulated over time, which is the basic limitation of the PDR system. However, as described in Section 3.3.2, the final position is calculated using PDR and the magnetic field data. So, the PDR data are used only for distance and heading estimation over a short period. Once the user location is finalized, PDR data are reset. It is superior to simple PDR and the gyro drift does not accumulate.

### 3.3. Localization Module

The localization module consists of two sub-modules: database formation and localization. A magnetic field pattern fingerprint database is made during the first sub-module, however, first, a compact analysis is made on the nature of magnetic field patterns from various smartphones.

#### 3.3.1. Database Formation

It has been already established that using the magnetic field data intensity as the fingerprint is not useful to devise an approach which can work with various smartphone in a similar fashion and provide similar localization performance, as the magnetic data intensity from various smartphone varies significantly. So many research works focus on the use of magnetic field data patterns as the fingerprint whereby the MP from one smartphone serves as the database and can be used for localization with different smartphones. It is assumed that the magnetic values though different but the shape/pattern of magnetic value is same/similar for different smartphones. This study first investigates this assumption and then presents an approach to utilize such magnetic field data patterns. Four similarity metrics have been used including Structural Similarity Index Measure (*SSIM*), Normalized Least Squared Error (*NLSE*), Root Mean Squared Error (*RMSE*), and Correlation (*CORR*) with the following formulas.

(14) $$\begin{array}{c} SSIM=\frac{\left(2\mu_{R}\mu_{I}+c_{1}\right)+\left(2\sigma_{R}\sigma_{I}+c_{2}\right)}{\left(\mu_{R}^{2}+\mu_{I}^{2}+c1\right)\left(\mu_{R}^{2}+\mu_{I}^{2}+c2\right)} \end{array}$$

(15) $$\begin{array}{c} NLSE=\sqrt{\frac{\sum_{m=1}^{M}\sum_{n=1}^{N}{\left[R\left(m,n\right)-I\left(m-n\right)\right]}^{2}}{\sum_{m=1}^{M}\sum_{n=1}^{N}{\left[R\left(m,n\right)\right]}^{2}}} \end{array}$$

(16) $$\begin{array}{c} RMSE=\sqrt{\frac{\sum_{m=1}^{M}\sum_{n=1}^{N}{\left[R\left(m,n\right)-I\left(m-n\right)\right]}^{2}}{MN}} \end{array}$$

(17) $$\begin{array}{c} CORR=\frac{2\sum_{m=1}^{M}\sum_{n=1}^{N}R\left(m,n\right)I\left(m-n\right)}{\sum_{m=1}^{M}\sum_{n=1}^{N}{\left[R\left(m,n\right)\right]}^{2}+\sum_{m=1}^{M}\sum_{n=1}^{N}{\left[I\left(m,n\right)\right]}^{2}} \end{array}$$

**Figure 6:**
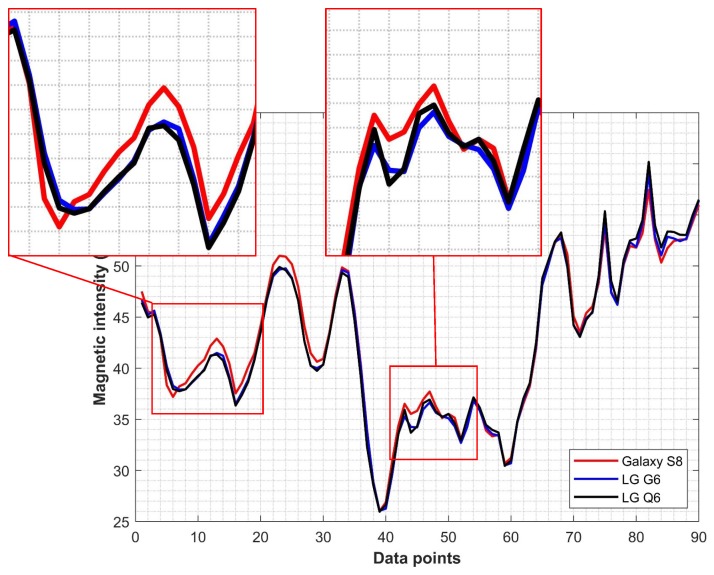
Magnetic field data patterns from Galaxy S8 and LG G6 for the same location.

Selected similarity metrics are applied to the data from Galaxy S8, LG G6, and LG Q6 as shown in Figure 6. The objective is to analyze the similarity of the magnetic field data from various smartphones. The data from different smartphones look very similar, however, when magnified as shown in two enlarged portions of Figure 6 the magnetic field data patterns are quite different. It becomes clearer when we use similarity measurement metrics.

To measure the similarity metrics, the data from Galaxy S8 is taken as the reference data and similarity values are calculated for LG G6 and Q6 data. The values for similarity metrics are shown in Table 2, where ‘value 1’ is for G6 data and ‘value 2’ for Q6 data. The values from similarity metrics indicate that the magnetic field data patterns are not the same for various smartphones. An estimated 20% to 25% magnetic field data patterns are different in shape based on the similarity metrics. These deviations may be different depending upon the smartphone data that is selected for comparison, however, the underlying theme is that the magnetic field data patterns for various smartphones are not the same, hence it is not appropriate to use the magnetic field data patterns from a single smartphone to prepare the fingerprint database.

**Figure 7:**
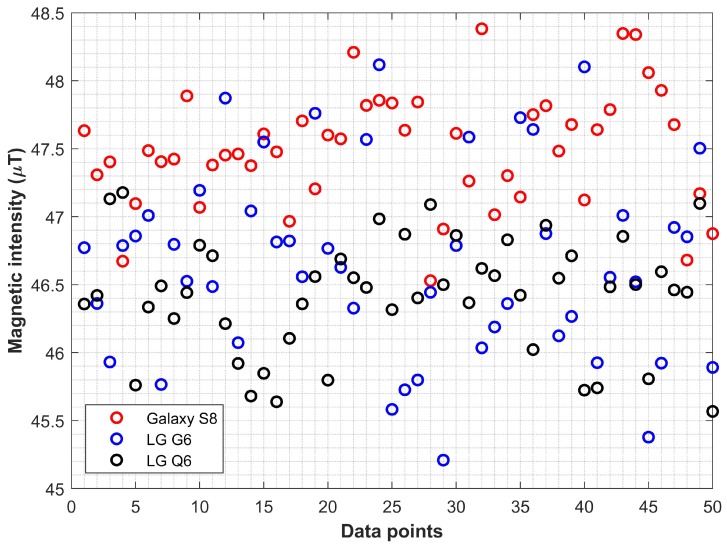
The magnetic field data collected for the same location using three different smartphones.

This study proposes the use of data from multiple smartphones to make the magnetic field data pattern database. Algorithm 1 is proposed to make the database. It aims to consolidate the magnetic data from multiple smartphones such that the outliers can be detected and removed and only the data concentrated around a centroid can be normalized to formulate the database. The reason to consider the outlier removal is the nature of collected data from a smartphone as shown in Figure 7. It is needless to say that the magnitude of data from the magnetometer of the same smartphone is different even for the very same location when the user is standing. A very slight movement of the user’s hand can change the magnitude of the magnetic field data. That is the reason that often multiple samples of magnetic field data are collected for the same location and normalized to overcome this issue.

Figure 7 shows the data from three smartphones collected for the very same location. The *x*-axis shows the number of samples while the *y*-axis represents magnetic field data intensity in μT. Since the data are scattered, so the first task in Algorithm 1 is to find the outliers and remove them.
**Algorithm 1** Make Magnetic Pattern Database. **Input:** Location coordinate L and magnetic data $M_{d}$ for three smartphones **Output:** Binary Grid (BG)- magnetic pattern database1:**for**i⟵1toL**do**2: $c⟵applyTwoSidedWindow(i,M_{d},k)$ // *k* represents the no. of subsequent and previous data points taken.3: $M_{df}⟵removeOutlier\left(c,\epsilon ,M_{d}\right)$4: $M_{dn}⟵normalizeData\left(M_{df}\right)$5:**end for**6:$BG⟵makeBinaryGrid(M_{dn}$

Algorithm 1 runs for all the location points L for which the magnetic field data $M_{d}$ are collected where the $M_{d}$ represents the data from Galaxy S8, LG G6 and LG Q6 for the current study. Location points L refers to all locations that are used to make the database and are separated by 1 m in a grid form. As shown in Figure 7 the data at any location $L_{i}$ can vary in magnitude even for the same smartphone, so, data normalization is essential to make the database. When sensor readings are projected over time, the problem of outlier detection is reduced to time series. Various approaches are available for time series outlier detection like z-score, neural networks, isolation forest, and window-based outlier detection [42]. We select the window-based outlier detection for its simplicity of implementation and efficiency. We have implemented a two-sided window neighbor that utilizes previous and subsequent data points to decide the outlier. Given the sensor reading time series $T_{s}=\langle d_{1}=\left(v_{1},t_{1}\right),d_{2}=\left(v_{2},t_{2}\right),\ldots ,d_{2}=\left(v_{n},t_{n}\right)\rangle$ where $v_{i}$ shows sensor value at $t_{i}$, neighboring points with two-sided windows are calculated as follows:

(18) $$\eta_{i}^{\left(k\right)}=d_{i-k},\ldots d_{i-1},d_{i+1},\ldots d_{i+k}$$

**Figure 8:**
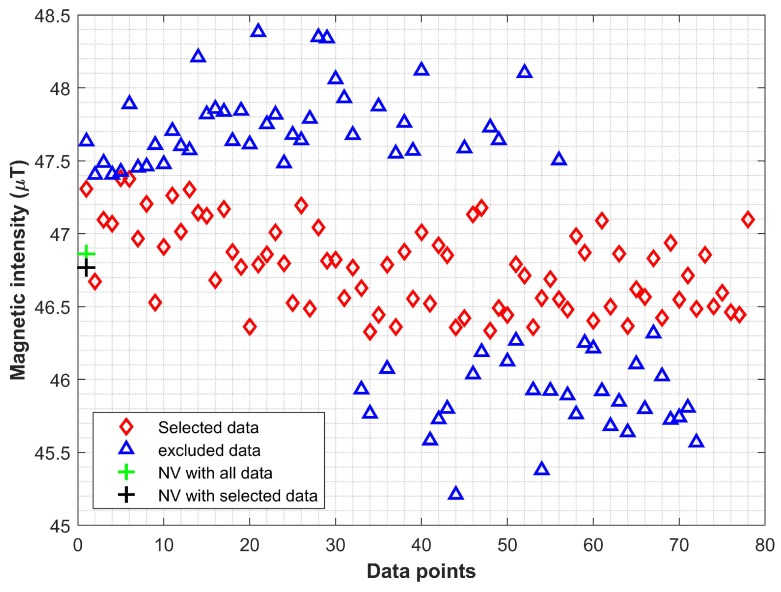
The normalization process of the magnetic data using Algorithm 1.

After calculating η, the outliers can be identified and removed using

(19) $$\left\{\begin{array}{cc} M_{d_{i}}\leq \left(\hat{\eta }+\epsilon \right) & select\\ otherwise & discard \end{array}\right.$$

where ϵ is the error margin considered to select the data for the database. As stated previously, the data for the same smartphone varies so we need to define an error margin (threshold) to filter out the outliers. The value of ϵ is empirically set to 0.50 μT and based on the observed variation in the collected magnetic field data. The data whose value is higher than c+ϵ are regarded as outliers and discarded as shown in Figure 8.

Once the outliers have been removed from the magnetic field data of the three smartphones, it can now be normalized (line 3 of Algorithm 1) using

(20) $$M_{dn}=\frac{1}{m}\sum_{i=1}^{m}M_{df}$$

where *m* represents the total number of filtered magnetic field data samples $M_{df}$. The normalization is done to get a stable value and a predominant method to make fingerprint database for magnetic field data positioning and localization systems.

After the normalization, the magnetic field data is transformed into the MP using the algorithm proposed in [14]. The transformed MPs serve as the database which is then used to perform indoor localization.

#### 3.3.2. Indoor Localization

**Figure 9:**
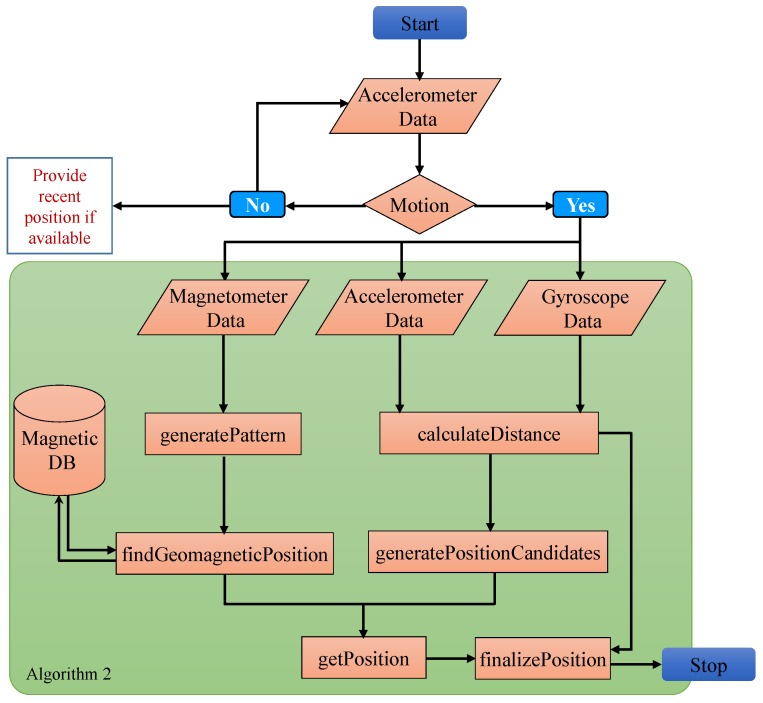
Flow chart of the approach used for user location estimation. Green shaded area shows the tasks carried out in Algorithm 2.

The localization process involves the use of the user collected data to estimate the location of the user. It is done using our approach previously presented in [14] and described here for completeness. The flow chart of the approach is shown in Figure 9.

**Figure 10:**
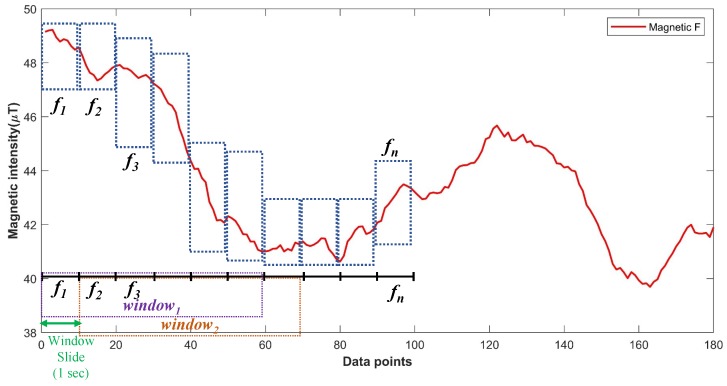
Explanation of ‘frame’ and ‘window’. Window sliding means a shift of one frame from the previous window.

User location is estimated using Algorithm 2 that takes the magnetic, accelerometer, and gyroscope data from smartphone sensors. Algorithm 2 uses *A* as the acceleration data, $D_{a}$ as the distance calculated using the acceleration data *A*, *G* as the magnetic data, *P* as set of positions, $P_{c}$ as set of candidate positions, $P_{g}$ as set of geomagnetic positions and $P_{f}$ as set of finalized positions.
**Algorithm 2** User Positioning Using Geomagnetic and Acceleration Data [14].1:**for**i⟵to5**do**2: **for**
j⟵toW
**do**3:  $D_{a}\left(j\right)⟵calculateDistance\left(A_{w}\right);$4:  $S\left(j\right)⟵generatePattern\left(G_{w}\right);$5:  $P_{g}\left(j\right)⟵findGeomagneticPosition\left(S\left(j\right)\right);$6: **end for**7: $P_{c}⟵generatePositionCandidates\left(P_{g},D_{a}\right);$8: $P_{i}⟵getPosition\left(P_{c},P_{g}\right);$9:**end for**10:$P_{f}⟵finalizePosition\left(P,D_{a}\right)$;

Other than that two key concepts to understand the working mechanism of the localization process are ‘frame’, and ‘window’ where the former refers to the data collected from smartphone sensors for 1 s at a sampling rate of 10 Hz while the latter represents ten consecutive frames. Window sliding is used by a shift of one frame as shown in Figure 10.

The localization process starts with the distance and heading estimation using the accelerometer and gyroscope data (line 3 of Algorithm 2), using the process described in Section 3.2. Then the magnetic field data is transformed into MP to estimate the position based on MP (line 4 of Algorithm 2). An initial magnetic field data based position is estimated using the ED between the user MP and the magnetic database. However, instead of taking only one position, we consider *n* positions for further processing where *n* is an empirical values and set to 10 (line 5 of Algorithm 2). Let the estimated positions for ten frames be $P_{g}=\{P_{g_{1}},P_{g_{2}},\ldots ,P_{g_{n}}\}$ and the distance calculated be $D_{a}=\{D_{a_{1}},D_{a_{2}},\ldots ,D_{a_{n}}\}$, the set of candidate positions can be calculated as

(21) $$P_{c_{n+1}}=P_{n}+D_{n+i}$$

(22) $$P_{c_{n-1}}=P_{n}-D_{n-i}$$

It means that if we know the current position of the user and the distance $d_{i}$ traveled by the user in time $t_{i}$ we can calculate user next position by taking current position and the distance. Conversely the previous position can be calculated by taking current position and distance $d_{i-1}$ traveled during $t_{i-1}$. Each estimated position in $P_{g}$ is considered as user true position and is used to define candidate positions using Equations (21) and (22) which yields ten sets of position candidates $P_{c}$ (line 7 of Algorithm 2). However, only one set is regarded as suitable to estimate user final position using

(23) $$P=\sqrt{\sum_{i=1}^{n}{\left(P_{gi}-P_{ci}\right)}^{2}}$$

Among the selected set of positions, the first element shows the starting position of the user. Five such windows are processed to calculate five positions. If five positions are not consistent, the outliers can be estimated and removed using

(24) $$V=|\frac{1}{n}\left(\sum_{i=1}^{n}\right)x_{i}-x_{i}|$$

(25) $$O=\left\{\begin{array}{c} 1,\;if\;V>\tau \\ 0,\;otherwise \end{array}\right.$$

where τ is the median value of two-sided window-based outlier function. It is calculated by taking previous and subsequent elements in the finalized positions using Equation (12). For example, if calculated positions for five windows be (1.48,21.1), (3.15,20.8), (31.84,21.0), (5.91,21.4) and (6.76,21.9), respectively, third position is an outlier in view of Equation (25). The outlier can be removed and the correct position can be estimated using the distance for that window. The positions corrected after the removal of outliers are called the finalized positions.

## 4. Experiments and Results

The first part of this section describes the experiment set up and the smartphones and their built-in sensors used for the experiments. Later, results of experiments focusing on the evaluation of various motion detection models and localization approach are carried out.

### 4.1. Experiment Set Up

Experiment is performed with four different smartphones including Samsung Galaxy S8, LG G6, LG G7, and LG Q6. The database (training data) is prepared with Galaxy S8, LG G6, and LG Q6 while the localization (testing) is done using all the four devices. It is important to point out that different datasets from S8, G6, and Q6 are used for training and testing. Table 3 shows the built-in sensors of the four smartphones that were used for the experiments.

### 4.2. Evaluation of Motion Detection Models

**Figure 11:**
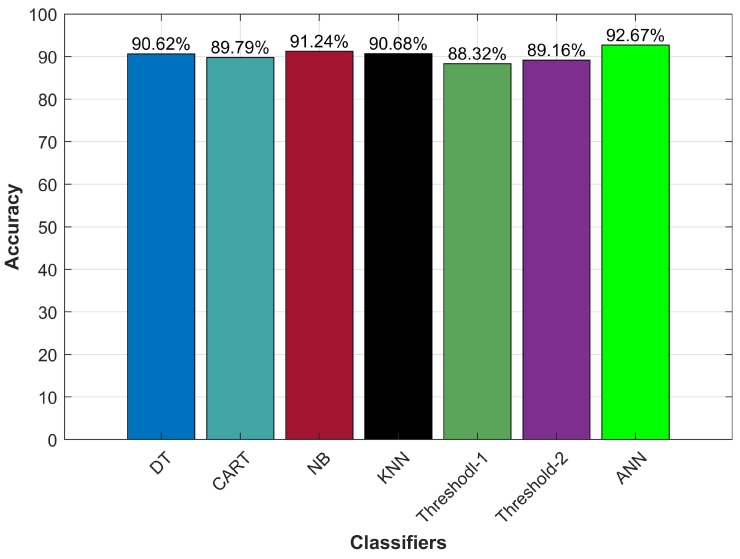
Motion detection accuracy with machine learning, threshold methods and ANN.

Motion detection involves the use of various algorithms to predict the user’s current states of stationary and walking which serves as an important module in indoor localization systems. It is important to detect if the user is walking or not. If the user is not walking then user new location estimation is not required which can save both energy and resources. Four machine learning classifiers and two methods that work on a threshold for acceleration are evaluated for their performance. Figure 11 shows the results for all the techniques.

Results demonstrate that ANN outperforms other machine learning, as well as, threshold-based methods to accurately classifying user’s state of walking and stationary. Research shows that the performance of ANN is better than that of traditional machine learning classifiers. The purpose of this experiment is to evaluate how closely the threshold methods can be to machine learning classifiers in terms of accuracy. It is important because although ANN has high accuracy yet it can not be deployed on smartphones. Instead, the location estimation procedure is carried out on the server-side and a communication link is needed between the user device and the server which increases the latency. On the other side, threshold methods do not require computational resources as does ANN, and hence are suitable to be used on the user device. The underlying purpose of this investigation is to investigate how accurate these threshold methods are, to be used as a motion detection tool.

There is no doubt that machine learning techniques perform better than that of threshold-based methods. However, threshold methods although not superior, can achieve accuracy very similar to that of other selected methods. Accuracy for $T_{1}$ and $T_{2}$ is 88.32% and 89.16% as against 92.67% of ANN’s. Threshold values for $T_{1}$ are 0.06, 0.10, 0.48, and 0.20 while for $T_{2}$ are 0.20, 0.15, 0.33, 0.34 for *x*, *y*, *z*, and *a* variances, respectively. The threshold methods do not require training like ANN does and can easily be deployed on user devices because they do not need high computing resources like the ANN. Hence they can reduce the latency and increase the performance of indoor localization systems.

#### Experiment Setup for Indoor Localization

**Figure 12:**
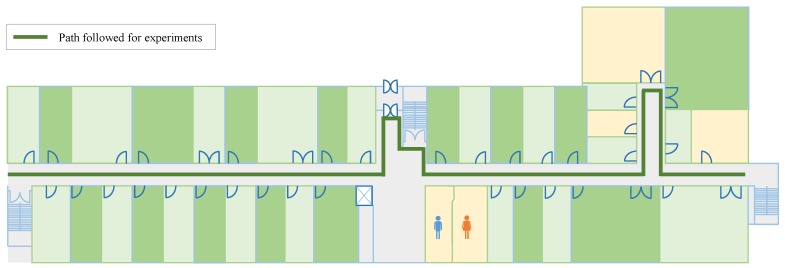
Path followed to perform indoor localization. The user walks along the same path with arbitrary direction.

Experiments are carried out in a University building to evaluate the performance of the proposed approach. The path followed for experiments is shown in Figure 12. Although the indoor environment is not a complex one, it is appropriate to evaluate the efficiency of the magnetic field based localization approach. The magnetic field data for the database are collected along the same path, yet in one direction only (from the left of Figure 12 to right).

### 4.3. Performance of Indoor Localization

Localization is performed using four devices: Galaxy S8, LG G6, LG G7, and LG Q6. The user can walk in any direction he wishes along the path shown in Figure 12 with the smartphone carrying in his hand. The starting position of the user is not known for the current approach and the user can select any random point to start with. The localization is performed at multiple days during a different time of the day for exhaustive results. Results are shown in Table 4 for at least 1250 location requests by the user for each smartphone. The error shown in Table 4 is calculated using:

(26) $$\sqrt{{\left(x_{p}-x_{g}\right)}^{2}+{\left(y_{p}-y_{g}\right)}^{2}}$$

where $x_{p}$ and $y_{p}$ are predicted while $x_{g}$ and $y_{g}$ are the ground truth values for user’s position.

Results demonstrate that the proposed approach works well to provide an accurate location of the user. The maximum error for any of the used smartphones is 7.56 m which is good considering that the user’s starting position is not known. Although Galaxy S8 performs exceptionally well, the accuracy of the other three smartphones is marginally different for mean and 50% errors. Error at 75% for LG Q6 is 4.05 which is the highest among all the smartphones. Galaxy S8, LG G6, and LG G7 can locate a user within 2.62 m, 3.25 m, and 2.74 m, respectively at 75%.

**Figure 13:**
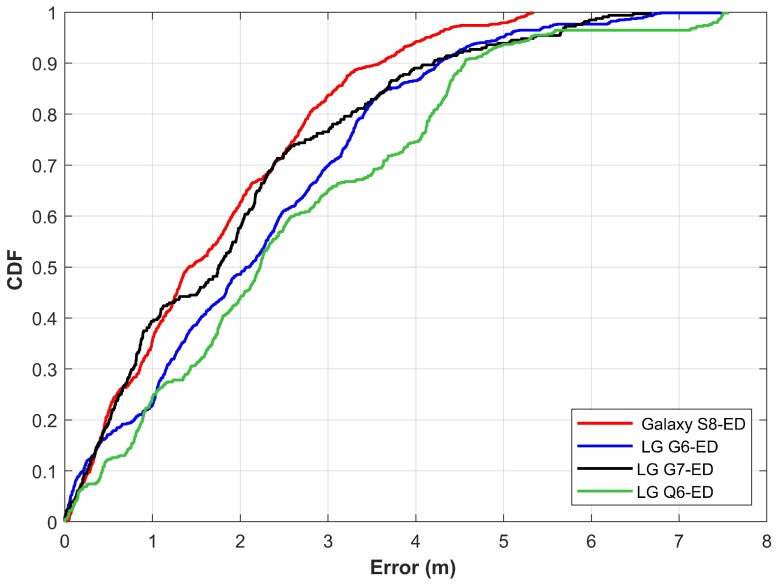
The CDF (cumulative Distributive Function) graph for localization using Galaxy S8, LG G6, LG G7 and LG Q6 using the proposed approach.

It is important to take into account the collective performance of the four smartphones used for experiments. The purpose of developing the database is to include the data from multiple devices to make the MP more effective when used with various smartphones for localization. The hypothesis was that it would increase the performance of the smartphones than that of using the database from a single smartphone. Results shown in Figure 13 prove the same. Results show that the localization performance with various smartphones, though marginally different, is almost identical. Another equally important point is the performance of LG G7. Even though the data from LG G7 were not incorporated in the database, the localization results of LG G7 are substantially similar to other devices.

The smaller differences in localization performance of various smartphones can be objected, but multiple factors should be considered. The MEMS sensors available in the selected smartphones are cheap and offer limited accuracy. Various smartphone companies and even various models of the same company may have different vendors’ sensors embedded in smartphones which makes it very hard to achieve the same localization performance with different smartphones. The magnetic field data from smartphone sensors are volatile and can be affected by the height of the user, phone position, and proximity of ferromagnetic materials. So, the localization performance is varied slightly even for the very same smartphone when used over different times and by different users. Keeping in view the above-mentioned factors, the performance of the current approach is quite promising.

### 4.4. Performance of Indoor Localization Using Dynamic Time Warping

The Euclidean distance is one of the widely used distance measurement techniques for indoor localization. However, Euclidean distance is not efficient with complex signal patterns/shapes [49]. Besides, the signal shape changes with smartphone sensors data in indoor localization due to the walking speed, height, and walking pattern of the users which reduces the localization performance. Dynamic Time Warping (DTW) can overcome such limitations by matching the corresponding points of two different length samples of data [50]. Originally designed for speech recognition systems, it can be applied to find similarity of time series data collected at various speeds [51]. Simple distance measures like euclidean distance follow a one-to-one linear alignment approach which the DTW is a non-linear one-to-many approach. For the current study, we use DTW with lower-bounding [52]. The procedure to estimate the user’s location is the same.

**Figure 14:**
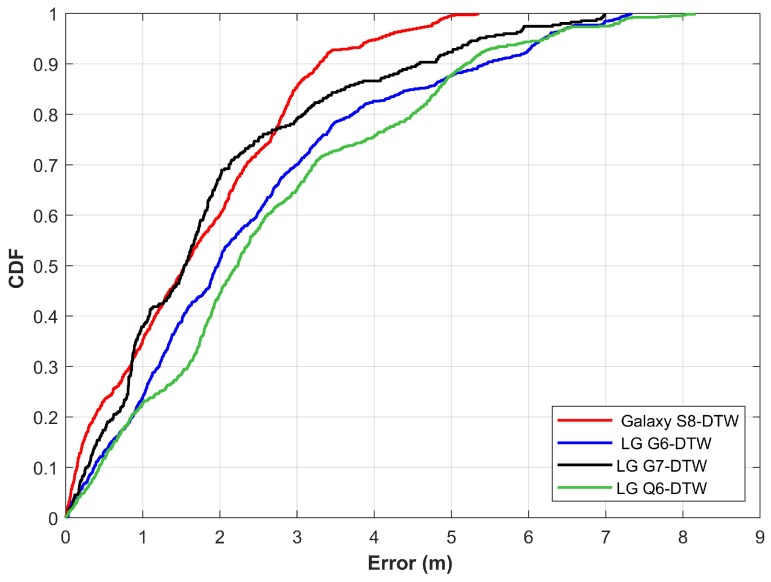
The CDF graph for localization results using dynamic time warping.

**Figure 15:**
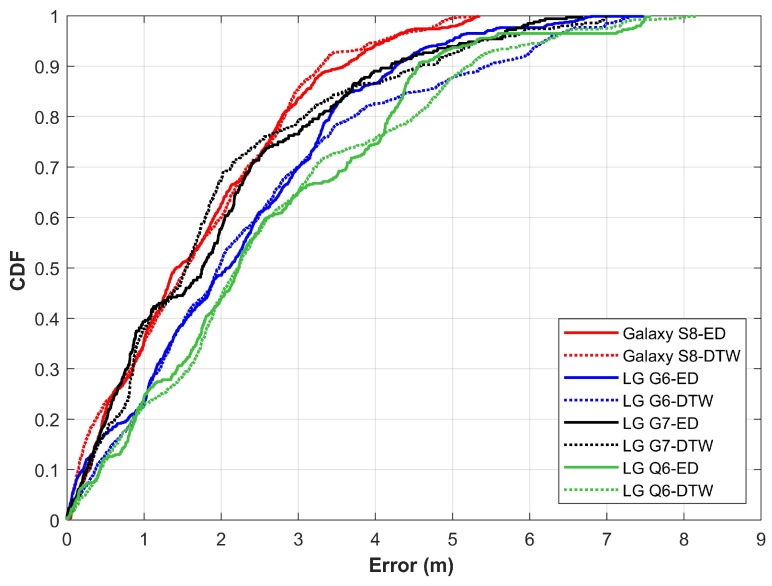
The comparison of localization results with euclidean distance and dynamic time warping.

Results shown in Figure 14 demonstrate the localization performance with DTW. Localization performance is approximately similar for all the smartphones when the DTW is used. Figure 15 gives the comparison of results for ED and DTW. The performance is slightly increased by using the DTW for matching the magnetic field patterns. It is due to the capability of DTW to match the data of different lengths which is not possible with ED.

Though the localization performance can be improved by selecting DTW over ED, the use of DTW is computationally expensive than that of ED. For example, the average time to calculate one position request using the DTW is 2.5141 s. However, if the ED is used, the average execution time is 1.852 s for the user’s single request. The results for minimum, maximum, mean, and second and third quartile are given in Table 5. These results also confirm that the use of the DTW has improved the localization results.

### 4.5. Performance Analysis with mPILOT [14]

The approach in this study is an extension of our previously published work [14], hence we compare the performance of both approaches and discuss the improvements. Table 6 shows the results from mPILOT and the current approach. There is no doubt that the current approach outperforms mPILOT. The mean, as well as, the maximum error has been reduced. Galaxy S8 mean error has been reduced to 1.54 m from 2.17 and LG G6 to 2.39 m from 2.96 m. There is a substantial improvement in the maximum error for both smartphones as well and it has reduced to 7.41 m from 11.69 m.

**Figure 16:**
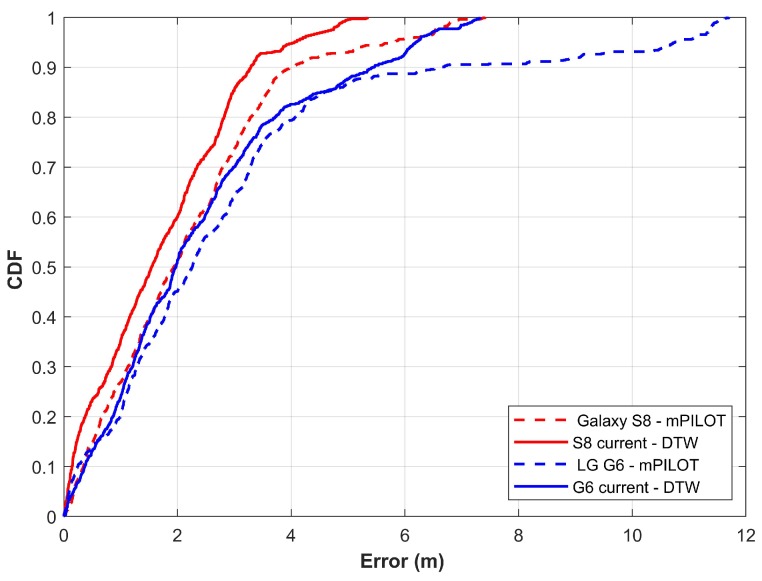
Performance comparison of current approach with mPILOT using Galaxy S8 and LG G6.

Results in Figure 16 indicate the enhanced performance of the current approach over mPILOT. LG G6’s performance is highly differentiated from Galaxy S8 with mPILOT. Now, it is not only improved but the performance is almost similar to Galaxy S8’s. The maximum error is minimized as well which shows that the current approach can mitigate the impact of using various smartphones on indoor localization. Thereby it is possible to achieve similar performance for indoor localization with different smartphones.

## 5. Discussions and Conclusions

Many magnetic field-based indoor localization approaches have been presented during the recent years, yet, the full potential of magnetic field-based localization systems is limited because a rich variety of smartphones collects the magnetic field data with different noise and sensitivity. Consequently, the localization performance varies significantly with various smartphones even when the very same localization approach is used. Although the use of Magnetic Pattern (MP) is proposed over the magnetic field data intensity to overcome the above-referred limitation, the localization performance is largely different from various smartphones as the MP is built from a single smartphone only. The current study analyzes the magnetic field data and reveals that the MP from various smartphones is not the same. So, this study proposes to use magnetic data from multiple smartphones to make the MP. An algorithm is presented which first identifies the outliers among the magnetic field data and removes them, and later normalizes the selected data to formulate the MP.

The localization approach is tested against four different smartphones including Galaxy S8, LG G6, LG G7, and LG Q6 with the MP that is made from S8, G6, and Q6. Results indicate that the use of MP from multiple smartphones produces localization results, though marginally different, yet almost identical from different smartphones. Additionally, the collective performance of four smartphones has improved as well. Performance comparison with other MP based approaches reveals that mean, 50%, and 75% error has been reduced. In addition to that, the maximum error has bee minimized to 7.47 m from 11.69 m from the compared approach. Besides, the study analyzes the impact of euclidean distance and dynamic time warping on localization accuracy. Results demonstrate that the DTW can elevate the localization accuracy than that of the ED, however, the processing time of DTW is higher. Currently, the localization is performed with only one orientation of the smartphone and the impact of change in user activities like phone listening and phone in pocket, etc. is left for future work.

## Figures and Tables

**Table 1:** Features used to train and test ANN for motion detection.

Feature	Description
varA	Variance in total acceleration
varAccX	Variance in acceleration of *x*-axis
varAccY	Variance in acceleration of *y*-axis
varAccZ	Variance in acceleration of *z*-axis

**Table 2:** Values for similarity metrics for given data.

Metric	Value 1	Value 2
*SSIM*	0.8390	0.8377
*NLSE*	0.1183	0.1243
*RMSE*	0.7723	0.7869
*CORR*	0.8889	0.8868

**Table 3:** List of the sensors contained in smartphones used for the experiment.

Sensor	Description
**Samsung Galaxy S8**	
SM-G950N Galaxy	S8 Octa-core, Adreno 540 GPU, Android 7.0 (Nougat), 4 GB RAM
Magnetometer (AK09916C)	3-axis, 16-bit, sensitivity 0.15 μT/LSB, temperature –30 to +85 ${}^{\circ }$C, 6.0 mA [43]
Accelerometer (LSM6DSL)	3-axis, 16-bit, sensitivity 0.061 mg/LSB, Temperature –40 to +85 ${}^{\circ }$C, 0.25 mA [44]
Gyroscope (LSM6DSL)	3-axis, 16-bit, sensitivity 125 mdps/LSB, Temperature –40 to +85 ${}^{\circ }$C, 6.1 mA
**LG G6**	
LGM-G600L LG	G6 Quad-core, Adreno 530 GPU, Android 7.0 (Nougat), 4 GB RAM
Magnetometer (AK09915C)	3-axis, 16-bit, sensitivity 0.15 μT/LSB, temperature –30 to +85 ${}^{\circ }$C, 6.0 mA [45]
Accelerometer (BMI-160)	3-axis, 16-bit, Temperature –40 to +85 ${}^{\circ }$C, 0.18mA
Gyroscoope (BMI-160)	3-axais, 16-bit, Temperature –40 to +85 ${}^{\circ }$C, 0.9 m
**LG G7**	
LM-G710N LG	G7 ThinQ Octa-core, Adreno 630 GPU, Android 9.0 (Pie), 4 GB RAM
Magnetometer (AK09918C)	3-axis, 16-bit, sensitivity 0.15 μT/LSB, temperature –30 to +85 ${}^{\circ }$C, 1.1 mA [46]
Accelerometer (IAM-20680)	3-axis, 16-bit, Temperature –40 to +85 ${}^{\circ }$C, 0.24mA [47]
Gyroscoope (IAM-20680)	3-axais, 16-bit, Temperature –40 to +85 ${}^{\circ }$C, 1.25 mA [47]
**LG Q6**	
LGM-X6OOS LG	Q6 Octa-core, Adreno 505 GPU, Android 7.1.1 (Nougat), 3 GB RAM
Magnetometer (AK09918C)	3-axis, 16-bit, sensitivity 0.15 μT/LSB, temperature –30 to +85 ${}^{\circ }$C, 1.1 mA [46]
Accelerometer (BMI-160)	3-axis, 16-bit, Temperature –40 to +85 ${}^{\circ }$C, 0.18mA [48]
Gyroscoope (BMI-160)	3-axais, 16-bit, Temperature –40 to +85 ${}^{\circ }$C, 0.9 mA [48]

**Table 4:** The summary of the results with all devices used for localization.

Device	Minimum	Maximum	Mean	50% Error	75% Error
Galaxy S8	0.006	5.35	1.72	1.48	2.62
LG G6	0.001	7.47	2.19	2.09	3.25
LG G7	0.003	6.67	1.93	1.75	2.74
LG Q6	0.002	7.56	2.53	2.23	4.05

**Table 5:** The summary of the results with all devices using dynamic time warping for localization.

Device	Minimum	Maximum	Mean	50% Error	75% Error
Galaxy S8	0.000	5.34	1.69	1.54	2.65
LG G6	0.005	7.32	2.39	1.97	3.27
LG G7	0.004	6.98	1.91	1.54	2.50
LG Q6	0.003	8.15	2.61	2.20	3.89

**Table 6:** Comparison of localization performance between mPILOT and the current approach.

Device-Technique	Mean	50% Error	75% Error	Maximum
S8-mPLIOT	2.17	1.93	3.01	7.41
S8-Current	1.54	1.54	2.65	5.34
G6-mPILOT	2.96	2.26	3.40	11.69
G6-Current	2.39	1.97	3.27	7.32
